# Little pigeons can carry great messages: potential distribution and ecology of *Uranotaenia* (*Pseudoficalbia*) *unguiculata* Edwards, 1913 (Diptera: Culicidae), a lesser-known mosquito species from the Western Palaearctic

**DOI:** 10.1186/s13071-017-2410-3

**Published:** 2017-10-10

**Authors:** Serhii Filatov

**Affiliations:** National Scientific Center Institute of Experimental and Clinical Veterinary Medicine, Kharkiv, Ukraine

**Keywords:** Culicidae, *Uranotaenia unguiculata*, Modelling, Potential distribution, Ecology, Vector-borne diseases

## Abstract

**Background:**

*Uranotaenia unguiculata* is a Palaearctic mosquito species with poorly known distribution and ecology. This study is aimed at filling the gap in our understanding of the species potential distribution and its environmental requirements through a species distribution modelling (SDM) exercise. Furthermore, aspects of the mosquito ecology that may be relevant to the epidemiology of certain zoonotic vector-borne diseases in Europe are discussed.

**Results:**

A maximum entropy (Maxent) modelling approach has been applied to predict the potential distribution of *Ur. unguiculata* in the Western Palaearctic. Along with the high accuracy and predictive power, the model reflects well the known species distribution and predicts as highly suitable some areas where the occurrence of the species is hitherto unknown.

**Conclusions:**

To our knowledge, the potential distribution of a mosquito species from the genus *Uranotaenia* is modelled for the first time. Provided that *Ur. unguiculata* is a widely-distributed species, and some pathogens of zoonotic concern have been detected in this mosquito on several occasions, the question regarding its host associations and possible epidemiological role warrants further investigation.

**Electronic supplementary material:**

The online version of this article (10.1186/s13071-017-2410-3) contains supplementary material, which is available to authorized users.

## Background

*Uranotaenia unguiculata* is the only species of the tribe Uranotaeniini Lahille, 1904 presently known to occur in Europe. Originally described from the Lake Kinneret area in Israel [1], the species for a long time was considered as a single representative of the genus in the entire Palaearctic region. More recently, Harbach & Schnur [2] reported the presence of another species, *Ur.* (*Pseudoficalbia*) *mashonaensis* Theobald, 1901, in the same area in Israel. Interestingly, this finding highlights that this group of mosquitoes is grossly understudied in the region.

Compared with the other, better-studied species of the Culicidae, very little is known about bionomics of *Ur. unguiculata* throughout its range. Immature stages of the species can be found from May to October across a variety of habitats, both in natural and urbanized areas [3–5]. Thus, larvae occur mostly in stagnant permanent, fresh or slightly brackish (up to 1000–1100 mg/l) water bodies, often overgrown with higher aquatic vegetation [5–8]; occasionally, they have also been found in more ephemeral environments such as rice paddies, puddles, hoof prints, and artificial containers [3, 9, 10]. Associations with instars of the following mosquito species have been recorded in the literature: *Anopheles algeriensis*, *An. hyrcanus*, *An. maculipennis* (*s*.*l*.), *An. sacharovi*, *An. sergentii*, *An. superpictus*, *Culex antennatus*, *Cx. judaicus*, *Cx. laticinctus*, *Cx. mimeticus*, *Cx. modestus*, *Cx. pipiens pipiens*, *Cx. perexiguus*, *Cx. theileri*, *Cx. univittatus*, *Culiseta alaskaensis*, *Cs. longiareolata*, *Cs. subochrea*, and *Ochlerotatus caspius* [3, 5–8, 11–16], which evidently suggest a broad ecological plasticity of *Ur. unguiculata*.

Even less is known regarding the ecology of adult stages, including the preferred host range, seasonal and daily activity patterns, and dispersal capabilities. Thus, *Ur. unguiculata* has repeatedly been referred to as an ornithophilic mosquito [17, 18] or feeding on amphibian and reptilian hosts has been suggested for the species, largely by the analogy with its congeners [5]. From this point of view, it is interesting to note that the females are attracted to light traps, which are routinely supplemented with carbon dioxide as a bait under the current mosquito surveillance schemes in Europe (e.g. [19, 20]). Although these data are rather inconclusive, with regard to the nature of this attraction, as for example, both sexes can be attracted to black light alone (SF, unpublished observations), at least in two instances female specimens of *Ur. unguiculata* were collected in traps using CO_2_ only [20, 21]. Independently from the used methods, the species is rarely collected in sufficient quantities, which usually leads authors to exclude it from any further ecological analysis (e.g. [22]). According to limited field observations, *Ur. unguiculata* presumably is a multivoltine mosquito [23], overwintering as unfed, inseminated, nulliparous females, usually in human-made shelters such as cellars, or in reed (*Phragmites* spp.) piles and under dense marsh vegetation in natural conditions [24–26]. Autogenous egg production has also been observed in some populations of this species [27, 28].

Although it has been widely recognized that *Uranotaenia* spp. are challenging to sample, especially in the adult stage [29–32], the lack of knowledge regarding ecology and distribution of these mosquitoes might have its consequences for our understanding of some vector-borne diseases that pose a threat to animal and human health. Namely, several strains of West Nile virus (WNV) (*Flaviviridae*: *Flavivirus*) have been repeatedly isolated from *Ur. unguiculata* in different parts of Europe [33–36] and a hypothesis regarding the possible role of the species in the virus overwintering has been suggested [26].

Modelling suitable habitats for arthropod vectors of human and animal diseases is a growing application of species distribution modelling (SDM) which helps in elucidating species-environment relationships and therefore can be used to support epidemiological studies or target disease surveillance [37–40]. A variety of methods such as Maximum Entropy (Maxent), Boosted Regression Trees (BRT), and Genetic Algorithm for Rule-set Production (GARP) have been widely explored and implemented to construct SDMs for mosquito vectors over the last decade [41–48]. Given the scarcity of information regarding the distribution and ecology of *Ur. unguiculata* across its range, it has been decided to model a potential distribution of the species with the aid of Maxent, which is a popular, user-friendly tool for predicting species distributions. Moreover, other aspects of the species fundamental niche such as trophic interactions and their potential consequences for the epidemiology of vector-borne diseases are being discussed in the present communication.

## Methods

### Study area and distributional data

Contrary to the geographical extent accepted by some of the recent works on the zoogeography of the Western Palaearctic Culicidae (e.g. Porretta et al. [49]), in the scope of this study borders of the region are defined as reported by De Lattin [50]. This area encompasses Europe, North Africa and the Eastern Mediterranean, and a vast area of South-Central Asia that lies west of China and is relatively well delimited by high Central Asian mountain ranges such as the Tien Shan and Karakoram mountains from the south. Moreover, as the exact borders between the Palaearctic and neighbouring biogeographical realms are often considered arbitrary, and for the sake of utility, the broad rectangular area lying between latitude 20–60°N and longitude 20°W to 80°E has been chosen for the modelling exercise. Hence the study area encompasses the entire species range reported in the literature [5, 7, 51].

**Fig. 1:**
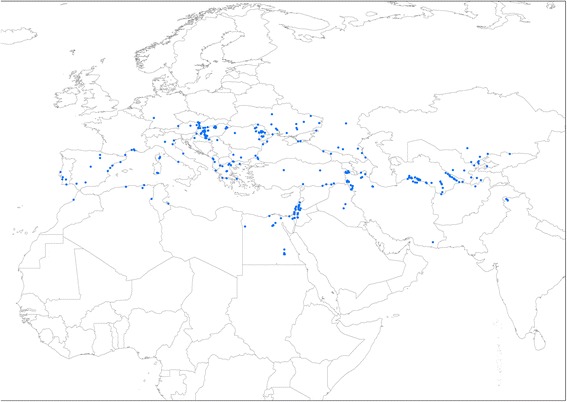
Distributional records of *Uranotaenia unguiculata* in the Western Palaearctic

Presence records for *Ur. unguiculata unguiculata* were compiled from a variety of sources, including review of the major literature on ecology and faunistics of Palaearctic Culicidae (see Additional file 1 for the literature search protocol); records held in Zoological Institute of the Russian Academy of Sciences, Saint Petersburg (A.V. Khalin, pers. comm.), Walter Reed Biosystematics Unit, Smithsonian Institution, Washington DC (accessed through the VectorMap data portal: http://www.vectormap.org, on 27 October 2015); through personal communications from a number of mosquito experts (see the Acknowledgements); as well as from the author’s own findings of the species in eastern and southern regions of Ukraine. For a substantial proportion of the data (mostly literature records) the exact presence points were unavailable and geographical coordinates were assigned using GeoLocate Web Application (http://www.museum.tulane.edu/geolocate/web/WebGeoref.aspx). Only localities that could be determined unambiguously were used for the georeferencing and included in the dataset, which consists of 308 spatially unique occurrence points (Fig. 1), spanning the period between 1913 and 2015. To augment availability of the distributional information, the data are provided as Additional file 2: Table S1.

### SDM development procedure

Maxent is a dedicated software based on the maximum-entropy approach for modelling species niches and distributions using their occurrence records together with a set of user-defined environmental predictor variables (i.e. temperature, precipitation) for a surrounding study area [52, 53]. This method was chosen because of its high performance, robustness, and, importantly, because of its ability to accurately predict species distributions from small numbers of occurrences [37, 54–56]. Briefly, Maxent predicts habitat suitability for a given species by comparing environmental predictors at sites where it has been recorded with background sites across the study area. The resulting (default) logistic output is visualized and interpreted as a habitat suitability index ranging from 0 (unsuitable) to 1 (highly suitable habitat) per grid cell [56–58].

Predictor variables used in the process of the model building must be ecologically relevant and ideally, as proximal as possible to the factors limiting the modelled species’ distribution [59]. A multitude of different environmental predictors has been used in SDM development for mosquitoes with temperature and precipitation variables (Bioclim) being the most widespread [41–47, 60–63]. Along with the climatic predictors, other types of variables such as elevation, land-cover, land use, and host population density have been complementary explored in some of these studies and found to be important contributors to the models [41, 45, 62]. However, as some of these variables (e.g. elevation) are distal to the true limiting factors [64] or largely are not available at broad spatial and temporal scales, it has been decided to use only Bioclim data as predictor variables in the present study. This dataset generated through interpolation of average monthly data from numerous meteorological stations over a 50-year period (1950–2000) [65] is available on a worldwide scale and broadly matches the temporal extent of the occurrence dataset for *Ur. unguiculata*.

Nineteen bioclimatic variables at a spatial resolution of 30 arc-seconds (~1 × 1 km^2^ grid cells) were downloaded from the WorldClim database (http://www.worldclim.org/bioclim), clipped to the study area extent and prepared for use in Maxent with the aid of the ArcMap 10.3.1 free extension toolkit SDM toolbox v.1.1c [66]. Subsequently, to be able to make inferences on the predictor variable importance, the environmental layers were checked for collinearity using the “Explore Climate: Correlations and Summary Stats tool” within the same toolkit and highly correlated (Pearson’s correlation coefficient > 0.80, see Additional file 3: Table S2) were removed from the list. A decision on whether to remove or retrieve a variable was also made on the basis of their percent contribution to the model obtained during a preliminary run in Maxent 3.3.k with the full set of environmental layers and presence records. The resulted list of predictors used for the final model development consisted of 11 variables (Table 1).

**Table 1:** Nineteen environmental variables obtained from the WorldClim dataset (http://worldclim.org)

Variable	Description
bio1	Annual mean temperature
bio2	Mean diurnal range^a^
bio3	Isothermality
bio4	Temperature seasonality
bio5	Maximum temperature of warmest month
bio6	Minimum temperature of coldest month^a^
bio7	Temperature annual range^a^
bio8	Mean temperature of wettest quarter^a^
bio9	Mean temperature of driest quarter
bio10	Mean temperature of warmest quarter^a^
bio11	Mean temperature of coldest quarter^a^
bio12	Annual precipitation^a^
bio13	Precipitation of wettest month
bio14	Precipitation of driest month
bio15	Precipitation seasonality
bio16	Precipitation of wettest quarter
bio17	Precipitation of driest quarter^a^
bio18	Precipitation of warmest quarter^a^
bio19	Precipitation of coldest quarter^a^

^a^Variables used in the analysis

Spatial clustering of occurrence points in some areas was evident in our dataset; and therefore, in order to avoid problems related to uneven sampling efforts and spatially autocorrelated records, which pertain to most if not all presence-only data gathered from museum records, online databases, etc. [67, 68], the original occurrence dataset was reduced within a specified Euclidean distance of 50 km (a distance exceeding the average maximum flight distance reported for the Culicidae [69]) using the “Spatially Rarefy Occurrence Data for SDMs” tool in the SDM toolbox. This resulted in a spatially rarefied dataset consisting of 151 occurrences, which was subsequently used for the modelling exercise.

For the final model run Maxent was set-up with the default settings except for the following: the regularization multiplier of 2, as the higher regularization parameter limits model complexity and reduce overfitting [70], and the random test percentage of 25 has been chosen as a simple test of model fit previously used by number of SDM studies for hematophagous vectors in the absence of independent data for model validation [40]. The resulted SDM was processed and visualized in the ArcMap 10.3.1.

As a measure of a model’s discriminative power Maxent calculates the area under the curve (AUC) for the receiver operation characteristic (ROC) score, which typically ranges between 0.5 (indicates no better than random prediction) and 1.0 (i.e. a perfect fit) [52, 71]. Additionally, to allow users to explore the importance of predictors, Maxent performs variable jackknifing, a procedure which builds multiple models excluding different variables and compares the models’ performance with and without each variable [72].

## Results

**Fig. 2:**
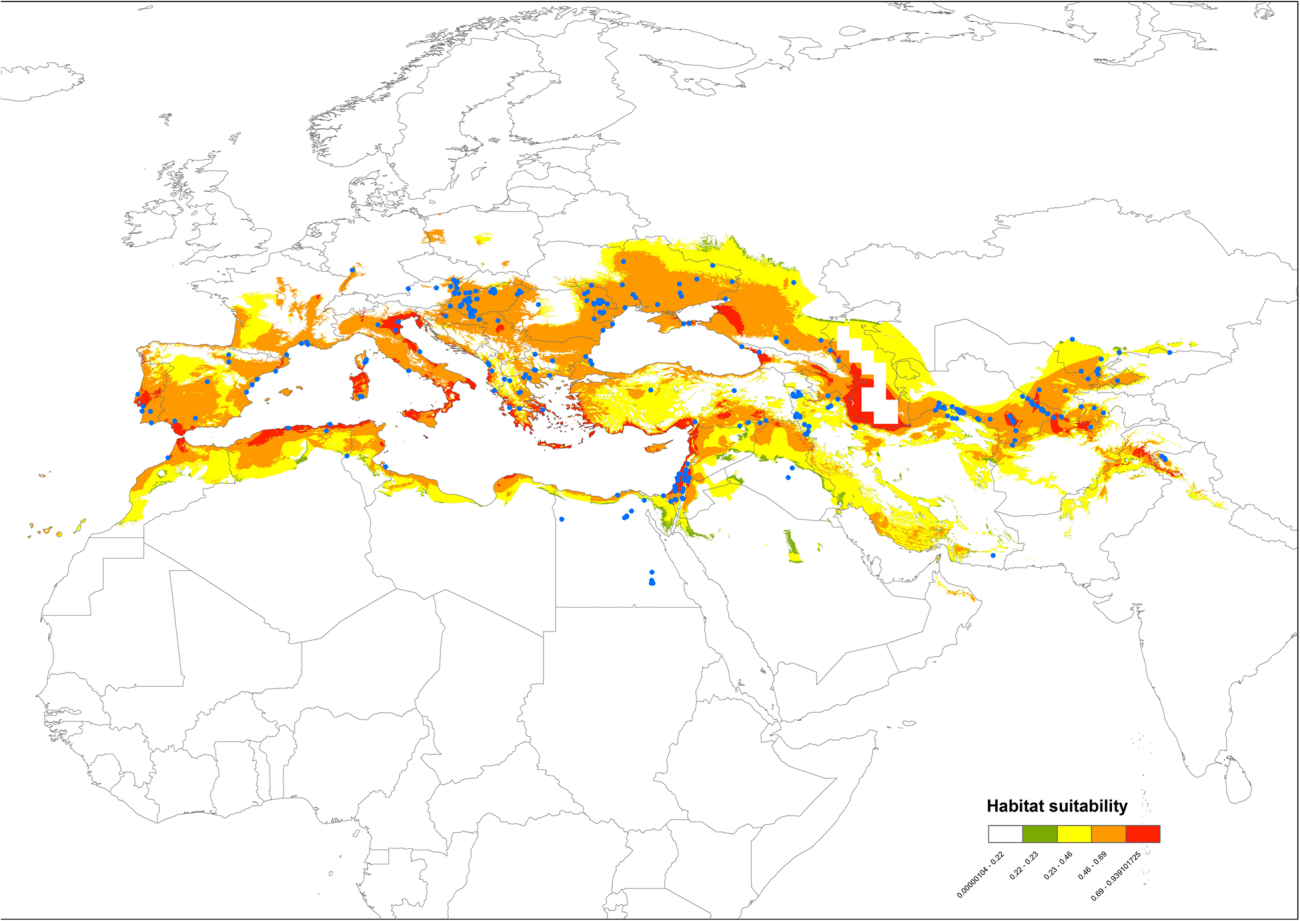
Model predictions for the potential distribution of *Uranotaenia unguiculata* in the Western Palaearctic together with the species occurrence records. The value of predicted environmental suitability was classified into five categories including very high suitability (*red*), high suitability (*orange*), moderate suitability (*yellow*), low suitability (*green*), and unsuitable (*white*)

Figure 2 shows model predictions for the potential distribution of *Ur. unguiculata* in the Western Palaearctic with the resulting area under the receiver operating characteristic (AUC) values of 0.913 and 0.882 ± 0.026 SD for the training and test data, respectively, which indicate high accuracy and predictive power of the model. Furthermore, the minimum training presence binomial probability of *P* < 0.001 shows that the model performance was better than random [73].

Summary data on the percent contribution and permutation importance for each environmental variable used in the analysis are presented in Table 2. Mean temperature of the warmest quarter (bio10) was a variable with highest percent contribution, followed by precipitation of the coldest quarter (bio19), and min temperature of the coldest month (bio6).

**Table 2:** Summary of the average percent contribution and permutation importance for each of the environmental variables

Variable	Percent contribution	Permutation importance
bio10	35.4	28.0
bio19	34.7	18.8
bio6	12.8	8.1
bio2	8.0	7.2
bio8	2.9	3.6
bio11	2.2	29.1
bio7	2.2	1.8
bio18	1.0	2.5
bio13	0.5	0.6
bio12	0.2	0.3
bio17	0.1	0

**Fig. 3:**
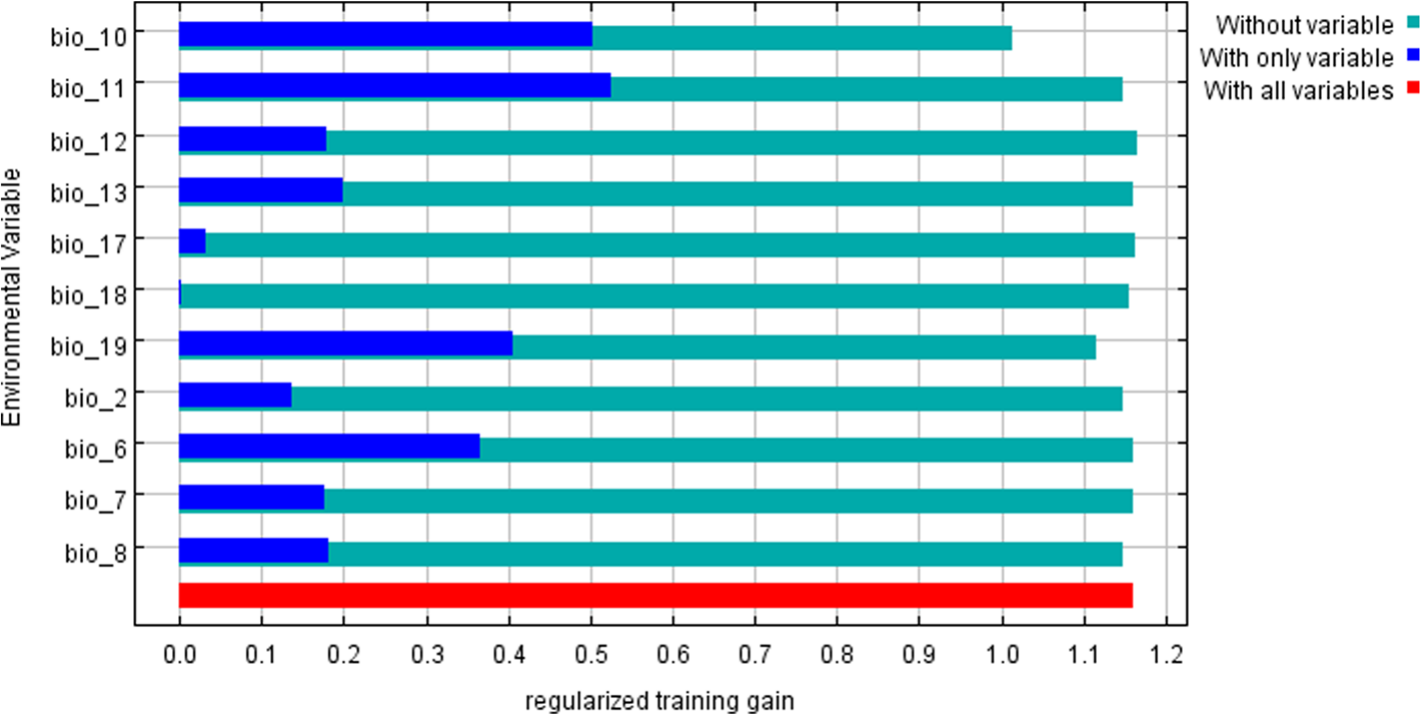
Jackknife of regularized training gain

According to the jackknife test (Fig. 3), mean temperature of the coldest quarter (bio11) was the variable having the highest training gain when used alone, which indicates that it has the most useful information by itself; whereas mean temperature of warmest quarter (bio 10) decreased training gain the most when was excluded from the analysis and therefore has the most information that is not present in other variables.

## Discussion

Although this study has several limitations resulting from the spatial and temporal biases and inaccuracies inherent in the types of data used in the analysis [74–76], the model is an appropriate first step to increase our understanding of the distribution of *Ur. unguiculata* [77, 78]. The known distribution of the species is well reflected by the model with only a few occurrence points falling outside the predicted range (Fig. 2). Moreover, most of the observed mismatches fall within the Saharo-Arabian subregion, a well-recognized biogeographical transition zone [79, 80] where at least two other *Uranotaenia* species have been reported to occur sympatrically with *Ur. unguiculata* [2, 81]. From this perspective, it is important to note that although the species is usually considered as immediately recognizable and easily identifiable throughout its range, it took some additional effort to realize that, for example, the specimens of *Uranotaenia* collected in Morocco belonged in the two separate taxa, a common Palaearctic *Ur. unguiculata* and an Afrotropical *Ur. balfouri* [81]. Hence, care should be taken when considering the records from areas with a strong influence of neighbouring biogeographical realms, which might represent misidentifications. The situation is further complicated by the fact that the subspecies *Ur. unguiculata pefflyi* has been described from the Arabian Peninsula [82], which renders the species polytypic. Given that the actual nature of this variability is essentially unknown, distributional data for *Ur. unguiculata pefflyi* were not included in the present analysis. The subspecific status of the Arabian populations could imply either classic allopatric speciation with niche conservatism, which would result in predicting these localities as suitable in the present model or intraspecific niche differentiation with poor habitat suitability predicted for the area [83, 84]. Albeit the data scarcity did not allow to model the distribution of this subspecies directly, the potential distribution of the nominal subspecies observed in the present study suggests the latter scenario. More information on the occurrence and preferably molecular data on the genetic variability of both subspecies in the region are needed to solve this problem.

The predicted areas of high habitat suitability within the European part of the range are in good agreement with known species records. At the same time, some areas in central and western Europe where occurrences for *Ur. unguiculata* are hitherto unknown surprisingly received a relatively high suitability index in the model. Thus, some areas in Poland (e.g. lower part of the River Oder basin in the Lubusz and Lower Silesian voivodeships, or even small coastal stretches on the Baltic shores near Kołobrzeg and Ustka located at some 54°N latitude!) represent suitable habitats according to the model results. Although it is unlikely that the species occurs so far north as the 54° latitude, it is reasonable to assume that the observed prediction reflects species’ true environmental requirements as inferred from the climatic variables, while it does not account for the array of other factors such as biotic interactions, climate equilibrium, and historical dispersal constraints [78, 85]. Similarly, the Canary Islands that have relatively poor mosquito fauna with only 11 species recorded so far [86] have been predicted by the model as largely suitable for *Ur. unguiculata,* although the archipelago is apparently unoccupied by the species, possibly as the result of its remoteness and recent volcanic origin*.*

Conversely, it has been conjectured that records of *Ur. unguiculata* from the Upper Rhine Valley in Germany [87] and the South Moravian Region of the Czech Republic [19] might represent a recent range expansion due to the global warming. However, as predicted by the model, these regions represent highly suitable areas and more likely serve as thermophilic refugia persisting at least since the Holocene climatic optimum [88], or even represent much older Pleistocenic extra-Mediterranean refugia, which have been increasingly recognized for various plant and animal taxa in Europe [89]. This should be unveiled with the new methods such as phylogenetic analysis of mtDNA sequencing data coupled with the species distribution models, which show great promise for understanding the patterns of biodiversity.

Although a full assessment of the species environmental requirements was behind the scope of the present study, the results of the jackknife test reveal interesting relationships between the mean temperatures of coldest (bio11) and warmest (bio10) quarters. The importance of winter temperatures as a factor limiting northerly distribution of temperate mosquitoes and expressed as a mean January isotherm has been elucidated for *Aedes albopictus* (reviewed in [90]), whereas sufficient summer temperatures are thought to be critical for immature development and the growth rates of mosquito populations [91]. It is possible that an interplay between the two variables is a factor that partially determines the presence of many thermophilic species in temperate latitudes, which is further supported by the notion that in areas “where 'summer' temperatures are high the effect of harsh 'winter' temperatures is less damaging” [92].

Interestingly, many of the species records utilized in the present analysis came from extensive wetland areas suggesting that the mosquito’s ecological niche might be overlapping with that of WNV in Europe [93]. Thus, the lineage 4 WNV has been detected in *Ur. unguiculata* pools from the Lower Volga region in Russia [33] and from mosquitoes collected in the Danube Delta, Romania [36]. Although the pathogenicity of the WNV strains isolated from *Ur. unguiculata* remains to be fully characterized and the species does not meet the classical criteria used to define a competent disease vector [94], further inquiries into the vector-pathogen-host interactions might prove useful for a better understanding of WNV evolution, routes of emergence and circulation in Europe. It is worth mentioning that currently *Ur. unguiculata* is rather perceived as an entomological curiosity among most European vector-borne disease specialists. However, this point of view is based on the species obscurity and a poor empirical knowledge of its ecology. One of the critical gaps is the lack of information regarding host preference and feeding behavior of the species. Indeed, although there is limited evidence in the literature that supports the notion that *Ur. unguiculata* feeds on the blood of poikilothermic animals [95–97], several field observations point out that the species do occasionally feed on mammalian hosts, including humans [98–100]. Together with the recent detection of canine parasitic nematodes *Dirofilaria repens* in pools of *Ur. unguiculata* from Moldova [101], this strongly suggests much broader host range for this mosquito species and its possible relevance to the circulation of zoonotic pathogens in Europe.

## Conclusions

To the best of the author’s knowledge, this study reports the first species distribution model for any of the representatives of the genus *Uranotaenia*. The results of the species distribution modelling exercise provide first insights into potential distribution and ecological requirements of *Ur. unguiculata* across the Western Palaearctic region. Provided that the species has a wide distribution and some pathogens of zoonotic concern have been detected in this mosquito on several occasions, the question regarding its host associations and possible epidemiological role warrants further investigation.

## Additional files

Additional file 1: Literature searches strategy and bibliography used to compile the occurrence dataset for *Uranotaenia unguiculata*. (PDF 666 kb)

Additional file 2: Table S1. Occurrence dataset for *Uranotaenia unguiculata*. (XLSX 29 kb)

Additional file 3: Table S2. Correlation matrix of the Bioclim environmental layers. Positive and negative correlations (≥0.80 or ≤ − 0.80) are shown in red. For variable names refer to the Table 1. (XLSX 12 kb)

## Abbreviations

BRT: Boosted Regression Trees
GARP: Genetic Algorithm for Rule-set Production
Maxent: Maximum Entropy
SDM: Species distribution modelling
WNV: West Nile virus
