# Supplementary material for: Little pigeons can carry great messages: potential distribution and ecology of Uranotaenia (Pseudoficalbia) unguiculata Edwards, 1913 (Diptera: Culicidae), a lesser-known mosquito species from the Western Palaearctic
Source: Parasit Vectors. 2017 Oct 10;10:464. doi: 10.1186/s13071-017-2410-3 (PMC5634949; doi:10.1186/s13071-017-2410-3)
Supplement: Supplementary file 1 — Literature searches strategy and bibliography used to compile the occurrence dataset for Uranotaenia unguiculata. (PDF 666 kb) [file 13071_2017_2410_MOESM1_ESM.pdf]

An exhaustive search of the peer-reviewed and 'grey' literature was undertaken with the search term "*Uranotaenia unguiculata*" in combination with the names of all countries within the species reported range (Ramsdale & Snow 2001) using Google Scholar and PubMed database. Additionally, references cited in these publications and in the faunistic monographs on Culicidae of the USSR were checked for the species records. The literature searches were performed until the 2nd of March 2016. Each potentially relevant source of distributional information was accessed in full text and checked against the next selection criteria:

- collection sites are reported at least on the level of the closest populated locality (e.g. village, town, etc.)
- life stages and identification methods of the collected mosquitoes are reported;

This resulted in the inclusion of 71 peer reviewed articles and books, as well as 5 qualification theses (listed below) to the final occurrence dataset.

Peer reviewed articles & books:

1. Stackelberg AA: **[Family Culicidae. Fauna of the USSR: Insecta, Diptera]**, vol. Vol. 3 Issue 4. Moscow: Nauka; 1937.
2. Sichinava S: **[Species composition of bloodsucking mosquitoes (Diptera, Culicidae) and their distribution according to topographical and climatic zones of Abkhazia]**. *Meditsinskaia parazitologiia i parazitarnye bolezni* 1972, **42**(3):313-315.
3. Fyodorova MV, Savage HM, Lopatina JV, Bulgakova TA, Ivanitsky AV, Platonova OV, Platonov AE: **Evaluation of potential West Nile virus vectors in Volgograd region, Russia, 2003 (Diptera: Culicidae): species composition, bloodmeal host utilization, and virus infection rates of mosquitoes**. *Journal of medical entomology* 2006, **43**(3):552-563.
4. Valkh SB: **[To the knowledge of Culicidae fauna in the eastern Ukraine]**. *Meditsinskaia parazitologiia i parazitarnye bolezni* 1959, **28**(6):687-695.
5. Volyanskaya EA: **[The presence of *Culex exilis* Dyar and *Uranotaenia unguiculata* Edw. in Odessa and Nikolaev Regions]**. *Meditsinskaya Parazitologiya i Parazitarnye Bolezni* 1958, **XXVII**(6):737.
6. Alekseev EV: **[On fauna of blood-sucking midges from the Kerch peninsula]**. *Vestnik zoologii* 1973(5):48-52.
7. Prendel ARA, S.S.; Korenchevskaya, G.A.: **[Mosquitoes of Nikolaev region and some territories contiguous to it]**. *Vestnik zoologii* 1967(4):71-73.
8. Shevchenko AK, Popovich AP, Steblyuk MV: **[Emergence Habitat of the Blood Suckling Mosquitoes in the Upper Part of Kakhovka Reservoir]**. *Vestnik zoologii* 1985(3):49-52.
9. Dolbeskin B: **Contribution a la faune des moustiques (Culicidae) du bassin du Dniepr (Ukraine)**. *CR Acad Sci USSR* 1928:229-232.
10. Rybinsky SB: **Matériaux Concernant La Faune des Moustiques de l'Ukraine du Nord-Ouest. I. — Les Moustiques du Polessié de l'Ukraine**. *Bulletin de la Société zoologique de France* 1933, **Séance du 10 janvier 1933**:18-29.
11. Rusev IT, Zacusilo VN, Vinnik VD: **Complex of fauna of mosquitoes in urban biocenosis and they role in circulation of west nile virus**. *Scientific Notes of Taurida VI Vernadsky National University – Series: Biology, chemistry* 2011, **24(63)**(2):240-248.

12. Sulesco TM, Toderas LG, Uspenskaia IG, Toderas I: **Larval Habitats Diversity and Distribution of the Mosquito (Diptera: Culicidae) Species in the Republic of Moldova**. *Journal of medical entomology* 2015, **52**(6):1299-1308.
13. Bitusik P, Hrabinova S: ***Uranotaenia unguiculata* (Diptera, Culicidae) - first record from Slovakia**. *Biologia, Bratislava* 1998, **53**(5):644.
14. Ungureanu EM: **Sur la presence d'*Uranotaenia unguiculata* Edwards dans la region du Nord de la Roumanie (Region de Iassy)**. *Archives Roumaines de Pathologie experimentale et de Microbiologie* 1942, **12**:475-477.
15. Mihályi F: **Igazi szúnyogok. Culicidae. [Mosquitoes. Culicidae]**. *Magyarország Állatvilága* 1955, **14**(5):1-40.
16. Kemenesi G, Dallos B, Oldal M, Kutas A, Földes F, Németh V, Reiter P, Bakonyi T, Bányai K, Jakab F: **Putative novel lineage of West Nile virus in *Uranotaenia unguiculata* mosquito, Hungary**. *Virusdisease* 2014, **25**(4):500-503.
17. Tóth S: **[Mosquito Fauna of Hungary]**. *Natura Somogyiensis* 2004, **6**:1-327.
18. Rudolf I, Šebesta O, Straková P, Betášová L, blažejová H, VEnclíková K, Seidel B, Tóth S, Hubálek Z, Schaffner F: **Overwintering of *Uranotaenia unguiculata* adult females in Central Europe: A possible way of persistence of the putative new Lineage of West Nile Virus?** *Journal of the American Mosquito Control Association* 2015, **31**(4):364-365.
19. Drenowsky AKI: **Notes sur l'anatomie, la morphologie et la biologie du moustique malarien et de sa larve. et II. La faune des moustiques des environs de Petritch.** . *Bulletin de la Direction de la santé publique en Bulgarie* 1929, **14 (Suppl. 2)**:118-137.
20. Božkov D, Hristova T, and Canev I: **Stechmücken an der bulgarischen Schwarzmeerküste**. *Bulletin de l'Institut de zoologie et musée,* 1969, **XXIX**:151-166.
21. Hristova T, I., Canev, and Smilova D: **[Changes in the species composition and distribution of the blood-sucking mosquitoes from the Bulgarian Black Sea coast]**. *Letopisi na HEI* 1971, **5**(31):174-183.
22. Ryba J, Hájková Z, Kaftan M: **Occurrence of Uranotaenia unguiculata Edwards, 1913 (Diptera, Culicidae) in Czechoslovakia**. *Folia parasitologica* 1973, **21**(2):142-142.
23. Šebesta O, Gelbič I, Minář J: **Mosquitoes (Diptera: Culicidae) of the Lower Dyje River Basin (Podyjí) at the Czech-Austrian border**. *Central European Journal of Biology* 2012, **7**(2):288-298.
24. Pachler K, Lebl K, Berer D, Rudolf I, Hubalek Z, Nowotny N: **Putative new West Nile Virus lineage in *Uranotaenia unguiculata* mosquitoes, Austria, 2013**. *Emerging Infectious Disease journal* 2014, **20**(12):2119.
25. Becker N, Kaiser A: **Die Culicidenvorkommen in den Rheinauen des Oberrheingebiets mit besonderer Berücksichtigung von *Uranotaenia* (Culicidae, Diptera) - einer neuen Stechmückengattung für Deutschland**. *Mitteilungen der Deutschen Gesellschaft für Allgemeine und Angewandte Entomologie* 1995, **10**(1-6):407-413.
26. Merdić E: **Mosquitoes (Diptera, Culicidae) of the park Maksimir, Zagreb, Croatia**. *Entomologia Croatica* 2002, **6**(1-2):51-56.
27. Bogojević MS, Hengl T, Merdić E: **Spatiotemporal monitoring of floodwater mosquito dispersal in Osijek, Croatia**. *Journal of the American Mosquito Control Association* 2007, **23**(2):99-108.
28. Boziclc B: **Investigation of mosquito fauna (Diptera, Culicidae) in Potisje**. *Tiscia (Szeged)* 1985, **20**:111-116.
29. Lytra I, Emmanouel N: **Study of *Culex tritaeniorhynchus* and species composition of mosquitoes in a rice field in Greece**. *Acta tropica* 2014, **134**:66-71.
30. Waterston J: **On the mosquitos of Macedonia**. *Bulletin of Entomological Research* 1918, **9**(1):1-12.
31. Pandazis G: **La faune des Culcides de Grèce**. *Acta Inst Mus Zool Univ Atheniensis* 1935, **1**:1-27.

32. Liberato CD, Magliano A, Farina F, Toma L: **Recent entomological enquiry on mosquito fauna in Circeo National Park**. *Annali dell'Istituto Superiore di Sanità* 2015, **51**(3):224-228.
33. Toma L, Cipriani M, Goffredo M, Romi R, Lelli R: **First report on entomological field activities for the surveillance of West Nile disease in Italy**. *Vet Ital* 2008, **44**(3):499-512.
34. Ascoli V, Facchinelli L, Valerio L, Zucchetto A, Dal Maso L, Coluzzi M: **Distribution of mosquito species in areas with high and low incidence of classic Kaposi's sarcoma and seroprevalence for HHV-8**. *Medical and veterinary entomology* 2006, **20**(2):198-208.
35. Aitken TH: **The Culicidae of Sardinia and Corsica (Diptera)**. *Bulletin of Entomological Research* 1954, **45**(3):437-494.
36. Ponçon N, Toty C, L'AMBERT G, Le Goff G, Brengues C, Schaffner F, Fontenille D: **Population dynamics of pest mosquitoes and potential malaria and West Nile virus vectors in relation to climatic factors and human activities in the Camargue, France**. *Medical and veterinary entomology* 2007, **21**(4):350-357.
37. DOBY J: **[New localities for some species of Culididae rarely noticed in France: *Uranotaenia unguiculata* Edwards 1913, *Culex impudicus* Ficalbi 1890, *Culex mimeticus* Noé 1899, *Theobaldia litorea* Shute 1928 and *Aedes longitubus* Cambournac 1938**. *Annales de parasitologie humaine et comparée* 1955, **30**(1-2):136.
38. Mouchet J, Rageau J: **Observations sur les moustiques I. L'hibernation d' *Uranotaenia unguiculata* Edwards, 1913 (Diptère Culicidae) De la Camargue et du Bas-Rhone** *Bull Soc Pathol Exot* 1965, **58**:246-250.
39. Bueno Marí R, Bernués Bañeres A, Chordá Olmos FA, Jiménez Peydró R: **Nuevos datos de *Uranotaenia unguiculata* Edwards, 1913 (Diptera: Culicidae) para la Península Ibérica**. *Boletín de la SEA* 2010(46):613-614.
40. Bueno Marí R, Rueda Sevilla J, Bernués Bañeres A, Lacomba Andueza I, Jiménez Peydró R: **Contribución al conocimiento de las poblaciones larvarias de culícidos (Diptera, Culicidae) presentes en el Marjal dels Moros (Valencia)**. *Boletín de la Asociación Española de Entomología* 2008, **32**(3-4):351-365.
41. Ferraguti M, Martínez-de la Puente J, Muñoz J, Roiz D, Ruiz S, Soriguer R, Figuerola J: **Avian Plasmodium in *Culex* and *Ochlerotatus* mosquitoes from southern Spain: effects of season and host-feeding source on parasite dynamics**. *PloS one* 2013, **8**(6):e66237.
42. Melero-Alcíbar R, Aranda C, Molina R: **Re-description of the pupa of *Uranotaenia unguiculata* (Diptera: Culicidae) and a morphological key for identification of pupae of mosquito genera in the Palaearctic Region**. *European Mosquito Bulletin* 2010, **28**:45-50.
43. Melero-Alcíbar R, Lucientes J, Molina R, Roiz D: **Revisión de *Uranotaenia (Pseudoficalbia) unguiculata* Edwards, 1913 en la Península Ibérica (Diptera: Culicidae)**. *Boletín de la SEA* 2005(36):347-348.
44. Torres Cañamares F: **Nota sobre tres Culícidos nuevos para España**. *EOS, Revista Española de Entomologia* 1944, **20**:65-70.
45. Almeida A, Galão R, Novo M, Sousa C, Parreira R, Pinto J, Carvalho L: **Update on the distribution of some mosquito (Diptera: Culicidae) species in Portugal**. *European Mosquito Bulletin* 2005, **19**:20-25.
46. Osório HC, Amaro F, Zé-Zé L, Moita S, Labuda M, Alves MJ: **Species composition and dynamics of adult mosquitoes of southern Portugal**. *Eur Mosquito Bull* 2008, **25**:12-23.
47. Ventim R, Ramos JA, Osório H, Lopes RJ, Pérez-Tris J, Mendes L: **Avian malaria infections in western European mosquitoes**. *Parasitol Res* 2012, **111**(2):637-645.
48. Ribeiro H, RAMOS HC, ALVES P, ANTUNES C: **Research on the mosquitoes of Portugal (Diptera, Culicidae). I. Four new culicine records**. *Anais do Instituto de Higiene e Medicina Tropical* 1977, **5**(1/4):203-214.

49. Edwards FW: **Tipulidae and Culicidae from the Lake of Tiberias and Damascus**. *Journal and Proceedings of the Asiatic Society of Bengal* 1913, **Vol. 9**(No. 1):47-51.
50. ŞİMŞEK FM: **Seasonal larval and adult population dynamics and breeding habitat diversity of *Culex theileri* Theobald, 1903 (Diptera: Culicidae) in the Gölbaşı district, Ankara, Turkey**. *Turkish Journal of Zoology* 2004, **28**(4):337-344.
51. Parr H: **The Culicine mosquitos of Syria and the Lebanon**. *Bulletin of Entomological Research* 1943, **34**(4):245-251.
52. ABDEL-MALEK AA: **The culicine mosquitoes of the northern region of the United Arab Republic**. *Bull Sot Entom Egypte* 1960, **44**:C1111.
53. Al-Khalili YH, Katbeh-Bader A, Amr Z: **Distribution and ecology of mosquito larvae in Jordan (Diptera: Culicidae)**. *Stud Dipterol* 2000, **7**(1):179-188.
54. Margalit J, Avrahami M, Tahori A: **Mosquitoes (Diptera: Culicidae) breeding in the Dead Sea Area**. *Israel Journal of Zoology* 1973, **22**(1):27-37.
55. Margalit J, Tahori A: **MOSQUITO SPECIES FOUND IN ISRAEL DURING A SURVEY 1955-58**. *Israel Journal of Entomology* 1970, **5**:151.
56. KHATTAT FH: **An Account of the Taxonomy and Biology of the Larvae of Cullcine Mosquitoes in Iraq. I. Central Iraq**. *Bulletin of endemic diseases* 1955, **1**(2):156-183.
57. Gad A: **Mosquitoes of the Oases of the Libyan Desert of Egypt**. *Bulletin de la Société entomologique d'Egypte* 1956, **40**.
58. Himmi O, Trari B, El Agbani MA, Dakki M: **Contribution à la connaissance de la cinétique et des cycles biologiques des moustiques (Diptera, Culicidae) dans la région de Rabat-Kénitra (Maroc)**. *Bulletin de l'Institut Scientifique, Rabat* 1998, **1997**(21):71-79.
59. Moussiegt O: **Les moustiques de Tunisie, leur répartition, bibliographie**. *Document EID* 1983, **47**:3-29.
60. Senevet G: **Notes sur les moustiques, 4-Quelques culicidés de la région de l'Aurès Algérie**. *Archives de l'Institut Pasteur d'Algérie* 1936, **14**(4):432-448.
61. Messai N, Berchi S, Boulknafd F, Louadi K: **Inventaire systématique et diversité biologique de Culicidae (Diptera: Nematocera) dans la région de Mila (Algérie)**. *Entomologie Faunistique-Faunistic Entomology* 2010.
62. Boudemagh N, Bendali-Saoudi F, Soltani N: **Inventory of Culicidae (Diptera: Nematocera) in the region of Collo (North-East Algeria)**. *Annals of Biological Research* 2013, **4**(3):1-6.
63. Barraud P: **The fauna of British India, Diptera Vol**. *V Family Culicidae Tribes Megarhinini and Culicini Taylor and Francis, London xxviii* 1934, **463**.
64. Balkashina EI: **La faune de Culicinae de la region du Kazakhstan mdridional.** . *Med Parazitol i Parazit Bol* 1939, **8**(5):19-38.
65. Kazantsev BN: **[Fauna of mosquitoes in Samarkand and adjacent regions]**. *Parasitol Sbornik ZIN AN SSSR* 1936, **6**:117-136.
66. Petrischeva PA: **Fauna, ecology and biology of Turkmenian Culicidae**. *Parazitologicheskiy Sbornik* 1936, **VI**:49-115.
67. Abai MR A-HS, Ladonni H, Hakimi M, Mashhadi-Esmail K, Sheikhzadeh K, Kousha A, Vatandoost H.: **Fauna and checklist of mosquitoes (Diptera: Culicidae) of East Azerbaijan Province, northwestern Iran.** *Journal of Arthropod-Borne Diseases* 2007, **1**(2):27-33.
68. Azari - Hamidian S, Yaghoobi - Ershadi MR, Javadian E, Abai MR, Mobedi I, Linton YM, Harbach RE: **Distribution and ecology of mosquitoes in a focus of dirofilariasis in northwestern Iran, with the first finding of filarial larvae in naturally infected local mosquitoes**. *Medical and Veterinary Entomology* 2009, **23**(2):111-121.

69. Azari Hamidian S, Joeafshani M, Mosslem M, Rassaei A: **Notes on Coquillettidia Richiardii and Uranotaenia Unguiculata (Diptera: Culicidae) in Guilan Province**. *Journal of Guilan University of Medical Sciences* 2004, **13**(51):1-9.
70. Azari-Hamidian S: **Larval habitat characteristics of mosquitoes of the genus *Culex* (Diptera: Culicidae) in Guilan Province, Iran**. *Journal of Arthropod-Borne Diseases* 2007, **1**(1):9-20.
71. S. Azari-Hamidian MRA, K. Arzamani, H. Bakhshi, H. Karami, H. Ladonni and R.E. Harbach: **Mosquitoes (Diptera: Culicidae) of North Khorasan Province, Northeastern Iran and the Zoogeographic Affinities of the Iranian and Middle Asian Mosquito Fauna**. *Journal of Entomology* 2011, **8**(3):204-217.

Qualification theses:

1. Gadzhieva SS: **[Avtoreferat: Fauna, biology, ecology of the genus *Anopheles* in the shore ecosystems of the Caspian sea]**. Dagestan, Makhachkala: Dagestan State University; 2010.
2. Prioteasa F-L: **Evaluarea potentialului vectorial al speciilor de Culicidae (Diptera-Insecta) din Delta Dunãrii, pentru virusul West-Nile (Flaviviridae)**. Universitatea din Bucuresti; 2011.
3. Koyumdzhieva M: **[A study of the larvae of non-malaric mosquitoes (tribus Culicini, subfam. Culicinae) in Sofia]**. Sofia: Sofia University; 1954.
4. Agushev T: **[Species composition, ecology and importance of the mosquito family (Culicidae) in the city of Plovdiv]**. Plovdiv: Agrarian University; 2015.
5. Rogozi E: **Bio-ecological and Taxonomical data on Culex mosquito genus (Diptera: Culicidae) in Albania**. Tirana: University of Tirana; 2013.
